# LGBTQ+ inequity in crowdfunding cancer costs: The influence of online reach and LGBTQ+ state policy

**DOI:** 10.1002/cam4.6926

**Published:** 2024-01-26

**Authors:** Austin R. Waters, Caleb W. Easterly, Cindy Turner, Lauren Ghazal, Ida Tovar, Megan Mulvaney, Matt Poquadeck, Stephen A. Rains, Kristin G. Cloyes, Anne C. Kirchhoff, Erin E. Kent, Echo L. Warner

**Affiliations:** ^1^ Department of Health Policy and Management, Gillings School of Global Public Health University of North Carolina Chapel Hill North Carolina USA; ^2^ Cancer Control and Population Sciences Huntsman Cancer Institute at the University of Utah Salt Lake City Utah USA; ^3^ College of Nursing University of Utah Salt Lake City Utah USA; ^4^ Crowdfunding Cancer Costs (C3) LGBTQ+ Study Advisory Board Huntsman Cancer Institute at the University of Utah Salt Lake City Utah USA; ^5^ School of Nursing University of Rochester Rochester New York USA; ^6^ School of Public Health Indiana University Bloomington Bloomington Indiana USA; ^7^ Wilmot Cancer Institute University of Rochester Medical Center Rochester New York USA; ^8^ Department of Communication University of Arizona Tucson Arizona USA; ^9^ School of Nursing Oregon Health and Science University Portland Oregon USA; ^10^ Department of Pediatrics University of Utah School of Medicine Salt Lake City Utah USA

**Keywords:** cost of care, crowdfunding, financial burden, financial hardship, financial toxicity, gender expansive, gender identity, sexual and gender minority, sexual minority, sexual orientation, transgender

## Abstract

**Background:**

Emerging literature suggests that LGBTQ+ cancer survivors are more likely to experience financial burden than non‐LGBTQ+ survivors. However, LGBTQ+ cancer survivors experience with cost‐coping behaviors such as crowdfunding is understudied.

**Methods:**

We aimed to assess LGBTQ+ inequity in cancer crowdfunding by combining community‐engaged and technology‐based methods. Crowdfunding campaigns were web‐scraped from GoFundMe and classified as cancer‐related and LGBTQ+ or non‐LGBTQ+ using term dictionaries. Bivariate analyses and generalized linear models were used to assess differential effects in total goal amount raised by LGBTQ+ status. Stratified models were run by online reach and LGBTQ+ inclusivity of state policy.

**Results:**

A total of *N* = 188,342 active cancer‐related crowdfunding campaigns were web‐scraped from GoFundMe in November 2022, of which *N* = 535 were LGBTQ+ and ranged from 2014 to 2022. In multivariable models of recent campaigns (2019–2022), LGBTQ+ campaigns raised $1608 (95% CI: −2139, −1077) less than non‐LGBTQ+ campaigns. LGBTQ+ campaigns with low (26–45 donors), moderate (46–87 donors), and high (88–240 donors) online reach raised on average $1152 (95% CI: −$1589, −$716), $1050 (95% CI: −$1737, −$364), and $2655 (95% CI: −$4312, −$998) less than non‐LGBTQ+ campaigns respectively. When stratified by LGBTQ+ inclusivity of state level policy states with anti‐LGBTQ+ policy/lacking equitable policy raised on average $1910 (95% CI: −2640, −1182) less than non‐LGBTQ+ campaigns from the same states.

**Conclusions and Relevance:**

Our findings revealed LGBTQ+ inequity in cancer‐related crowdfunding, suggesting that LGBTQ+ cancer survivors may be less able to address financial burden via crowdfunding in comparison to non‐LGBTQ+ cancer survivors—potentially widening existing economic inequities.

## 1 INTRODUCTION

In 2019, the projected patient economic burden of cancer in the United States, including out‐of‐pocket costs and time costs, was estimated to be $21.1 billion.^1^ A systematic review assessing the prevalence of cancer‐related financial burden found that nearly half of cancer survivors experienced some form of financial burden.^2^ Further recent literature suggests that the prevalence of financial burden among cancer survivors is likely even higher following the economic impact of the COVID‐19 lockdowns.^3^, ^4^

As a result of cancer‐related financial burden, survivors often engage in online crowdfunding on websites, such as GoFundMe.com, to raise money to pay for their healthcare and living expenses during and after treatment. It is estimated that 19 million crowdfunding campaigns are created each year, with one third of them focused on crowdfunding for medical costs.^5^ Medical crowdfunding success (often conceptualized as amount raised or percentage of goal raised) is associated with a variety of factors, including where the campaign was initiated (i.e., geographical location), the online reach of the campaign (e.g., shares, going viral, etc.), and content characteristics (e.g., third person campaign narrative).^6^, ^7^, ^8^ However, while crowdfunding among cancer survivors is common, as cancer campaigns are the most common type of medical campaign on GoFundMe.com, it has been found to widen existing economic inequities.^5^, ^9^ Inequity in crowdfunding can, in part, be explained by variation in the strength of existing social networks. Most crowdfunding campaigns do not go viral (i.e., quick and wide spread of information online), rather, they circulate within the cancer patient's social networks, thus subjugating the success of the campaign to underlying social and financial capacity of those within their network.^10^, ^11^

The lesbian, gay, bisexual, transgender, queer, and other sexual and gender minorities (LGBTQ+) population make up at least 7.1% of the US population.^12^ Individuals who identify as LGBTQ+ have nearly double the likelihood of living in poverty than non‐LGBTQ+ individuals (21.6% vs. 12.1%) and experience substantial employment discrimination, both of which contribute to economic instability among LGBTQ+ cancer survivors.^13^, ^14^, ^15^ Emerging literature suggests that LGBTQ+ cancer survivors may experience cancer‐related financial burden more frequently than their non‐LGBTQ+ counterparts.^16^, ^17^ Furthermore, due to limited collection of sexual orientation and gender identity (SOGI) data in national datasets, financial burden and the associated financial coping behaviors such as online crowdfunding have not been widely explored among LGBTQ+ cancer survivors. We addressed this gap by using novel community‐engaged and technology‐based methods to identify LGBTQ+ campaigns on GoFundMe.com and assess inequity in crowdfunding amounts raised between LGBTQ+ and non‐LGBTQ+ cancer crowdfunding campaigns.^18^

## 2 METHODS

In the crowdfunding cancer costs (C3) LGBTQ+ study, we integrated community‐engaged and technology‐based methods, including an LGBTQ+ study advisory board, web‐scraping, and identification of cancer‐related and LGBTQ+ crowdfunding campaigns using term dictionaries. These methods were integrated to enhance rigor and keep the LGBTQ+ community centered in our research by engaging our LGBTQ+ study advisory board to modify and apply term dictionaries to classify campaigns as LGBTQ+ versus non‐LGBTQ+ and cancer versus non‐cancer. A full description of this novel methodological approach is published elsewhere.^18^ This research was considered exempt from ethics approval by the University of Utah Institutional Review Board as it involved only publicly available data (IRB#00154744).

### 2.1 C3 LGBTQ+ study advisory board

The role of the LGBTQ+ study advisory board (SAB) was to co‐create knowledge about LGBTQ+ cancer crowdfunding by developing and implementing study methods and analyses as part of the study team. The study team consisted of SAB members and researchers from universities across the United States. Community‐engaged research principles that guided this process included prioritizing reciprocal mutually beneficial partnerships, SAB involvement at every stage of the research study, and continued engagement beyond study completion.^19^ The C3 LGBTQ+ SAB was initiated in May 2022. Individuals were eligible to join the SAB if they (1) self‐identified as LGBTQ+ and had a prior cancer diagnosis or cancer caregiving experience or (2) were a clinical professional working primarily with the LGBTQ+ community and an advocate for LGBTQ+ equity. Eight members were recruited from prior studies with LGBTQ+ cancer survivors and professional contacts of the study team resulting in a national board of primarily LGBTQ+ cancer survivors.

The SAB met four times over the course of 1 year to engage in a variety of study‐related activities including the development of the original LGBTQ+ term dictionary. The study team including the SAB developed and refined the LGBTQ+ term dictionary used to identify LGBTQ+ crowdfunding campaigns. The study team also modified Silver et al.'s cancer term dictionary to identify cancer‐related campaigns more accurately by removing terms that commonly led to misclassification.^9^, ^18^ The validity of term dictionaries was assessed through several rounds of blinded campaign classification coding completed by the study team and SAB members until acceptable pairwise agreement was reached.^18^

### 2.2 C3 LGBTQ+ dataset

Web‐scraping methods were used to collect all available health‐related campaigns from the sitemap GoFundMe.com to create the C3 LGBTQ+ dataset. Content that was collected via scraping included: campaign title, campaign description (including campaign updates), date created, city and state of campaign origin, goal amount, number of donors, and amount raised. The refined term dictionaries were applied to the crowdfunding dataset to identify cancer‐related and LGBTQ+‐related campaigns for analysis. The GoFundMe.com sitemap was initially accessed on November 11, 2022; web‐scraping was completed on November 22, 2022.

#### 2.2.1 Campaign characteristic variables

Campaigns were categorized by state into nine geographic locations using the US divisions as defined by the US Census Bureau, and campaigns originating outside the United States or with no campaign description in English were excluded.

A five‐level online reach variable was generated using quartiles of donors and was informed by the SPIN framework (Spreadability, Propagativity, Integration, and Nexus) of virality of information on social media.^20^ Number of donors was categorized as minimal, low, moderate, high, and viral online reach based on quartiles of number of donors and the median number of donors on crowdfunding websites in general (i.e., 47). The highest category was designated as viral online reach for a crowdfunding campaign based on propagativity of information online and the average offline social network size of individuals being less than 150 individuals.^21^ While online social networks may appear larger in number of “followers” or “friends” they often behave similarly to offline networks in terms of engagement.^22^

Lastly, informed by the Human Rights Campaign's (HRC) 2022 State Equality Index, we generated a categorical variable of LGBTQ+ inclusivity based on state‐level policy.^23^ Annually, the HRC scores each US state based on their LGBTQ+ policy across a set of domains; those reviewed in 2022 included parenting, relationship recognition and religious refusal, non‐discrimination, hate crimes and criminal justice, youth related, and health and safety. Each state is then scored on a four‐point scale (1 = high priority to achieve basic equality, 2 = building equality, 3 = solidifying equality, and 4 = working toward innovative equality.). To create our binary variable, states that were categorized as having anti‐LGBTQ+/lacking equitable policy (HRC score = 1) and states with some/all equitable policy (HRC score = 2–4).

Amount raised and goal amount were adjusted by year for inflation to 2022 dollars using the annual average Consumer Price Index All Urban, All Items US City Average.^24^

### 2.3 Statistical analyses

Descriptive statistics including mean, standard deviation (SD), and interquartile range (IQR) were calculated for LGBTQ+ and non‐LGBTQ+ campaigns including goal amount, number of donors, amount raised, year initiated, online reach, geographic location, and the HRC state policy index. Two‐way *t*‐tests were used to assess differences in campaign characteristics between LGBTQ+ cancer campaigns and non‐LGBTQ+ cancer campaigns. Prior to conducting *t*‐tests on goal amount and amount raised, values were log transformed to account for non‐normality. Number of donors, goal amount, and amount raised were then stratified by online reach and were again compared between LGBTQ+ and non‐LGBTQ+ campaigns using two‐way *t*‐tests. While data were log transformed prior to conducting two‐way *t*‐tests on goal amount and amount raised, untransformed 2022 dollars are reported in tables. Generalized linear models (GLMs) with a gamma distribution and log link were used to generate estimates of predicted amounts raised, differential effects, and 95% confidence intervals (95% CI) of LGBTQ+ status on amount raised by cancer campaigns. Stratified models were then run by online reach and HRC state policy index to explore differences in estimates. All models controlled for online reach, the US division, and year the campaign was initiated.

Due to unobserved factors that may vary between LGBTQ+ and non‐LGBTQ+ campaigns over time, such as deletions, the primary GLM models were conducted using 2019–2022 data while bivariate analyses and a sensitivity analysis were conducted using 2014–2022 data. The sensitivity analysis included identical GLM models that were run to explore how estimates changed when campaigns that have been open through unobserved factors that may vary by LGBTQ+ identification were included.

## 3 RESULTS

A total of *N* = 188,342 cancer‐related crowdfunding campaigns were active on GoFundMe.com as of November 22, 2022 (Table 1). Of those campaigns, *N* = 535 were identified as LGBTQ+ campaigns. LGBTQ+ campaigns had an average goal amount of $26,619 (median: $11,447; SD: 65,409; IQR: 6097–27,001), average number of donors of 110 (median: 59; SD: 177; IQR: 31–114), and raised an average of $10,991 (median: $5377; SD: 17,471; IQR: 2635–12,175). Non‐LGBTQ+ campaigns (*N* = 188,342) had an average goal amount of $66,577 (median: $11,939; SD: 6,216,105; IQR: 6096–25,000), average number of donors of 78 (median: 45; SD: 146; IQR: 26–87) and raised an average of $9194 (median: $4841; SD: 15,480; IQR: 2593–10,160).

**TABLE 1:** Cancer‐related crowdfunding campaign characteristics by LGBTQ+ status.

	LGBTQ+ campaigns (*N* = 535)	Non‐LGBTQ+ campaigns (*N* = 188,342)	*p*‐value
	Mean (SD) or *N* (%)	Mean (SD) or *N* (%)	
Number of donors	110 (177)	78 (146)	<0.0001
Goal amount ($)	26,619 (65,409)	66,577 (6,216,105)	0.86
Amount raised ($)	10,991 (17,471)	9194 (15,480)	0.19
Year initiated			
2014	12 (2.3)	5556 (2.9)	<0.0001
2015	17 (3.2)	12,770 (6.8)	
2016	24 (4.5)	14,051 (7.5)	
2017	32 (6.0)	20,111 (10.7)	
2018	57 (10.7)	22,664 (12.0)	
2019	71 (13.3)	22,334 (11.9)	
2020	75 (14.0)	24,170 (12.8)	
2021	110 (20.6)	30,746 (16.3)	
2022	136 (25.5)	35,883 (19.1)	
Online reach			
Minimal (3–25 donors)	102 (19.1)	46,448 (24.7)	<0.0001
Low (26–45 donors)	98 (18.3)	47,998 (25.5)	
Moderate (46–87 donors)	158 (29.5)	47,399 (25.2)	
High (88–240 donors)	130 (24.3)	37,094 (19.7)	
Viral (241–15,881 donors)	47 (8.8)	9403 (5.0)	
Geographic location			
New England	51 (9.5)	10,004 (5.3)	<0.0001
Middle Atlantic	63 (11.8)	21,052 (11.2)	
East North Central	63 (11.8)	24,869 (13.2)	
West North Central	23 (4.3)	14,155 (7.5)	
South Atlantic	87 (16.3)	38,174 (20.3)	
East South Central	17 (3.2)	8374 (4.5)	
West South Central	47 (8.8)	23,988 (12.7)	
Mountain	43 (8.0)	16,652 (8.8)	
Pacific	141 (26.4)	31,074 (16.5)	
LGBTQ+ inclusivity of state policy			
Some/all equitable policy	367 (68.6)	88,205 (46.8)	<0.0001
Anti‐LGBTQ+ policy/lacking equitable policy	168 (31.4)	100,137 (53.2)	

*Note*: All dollar amounts were adjusted for inflation to 2022 dollars.

**FIGURE 1:**
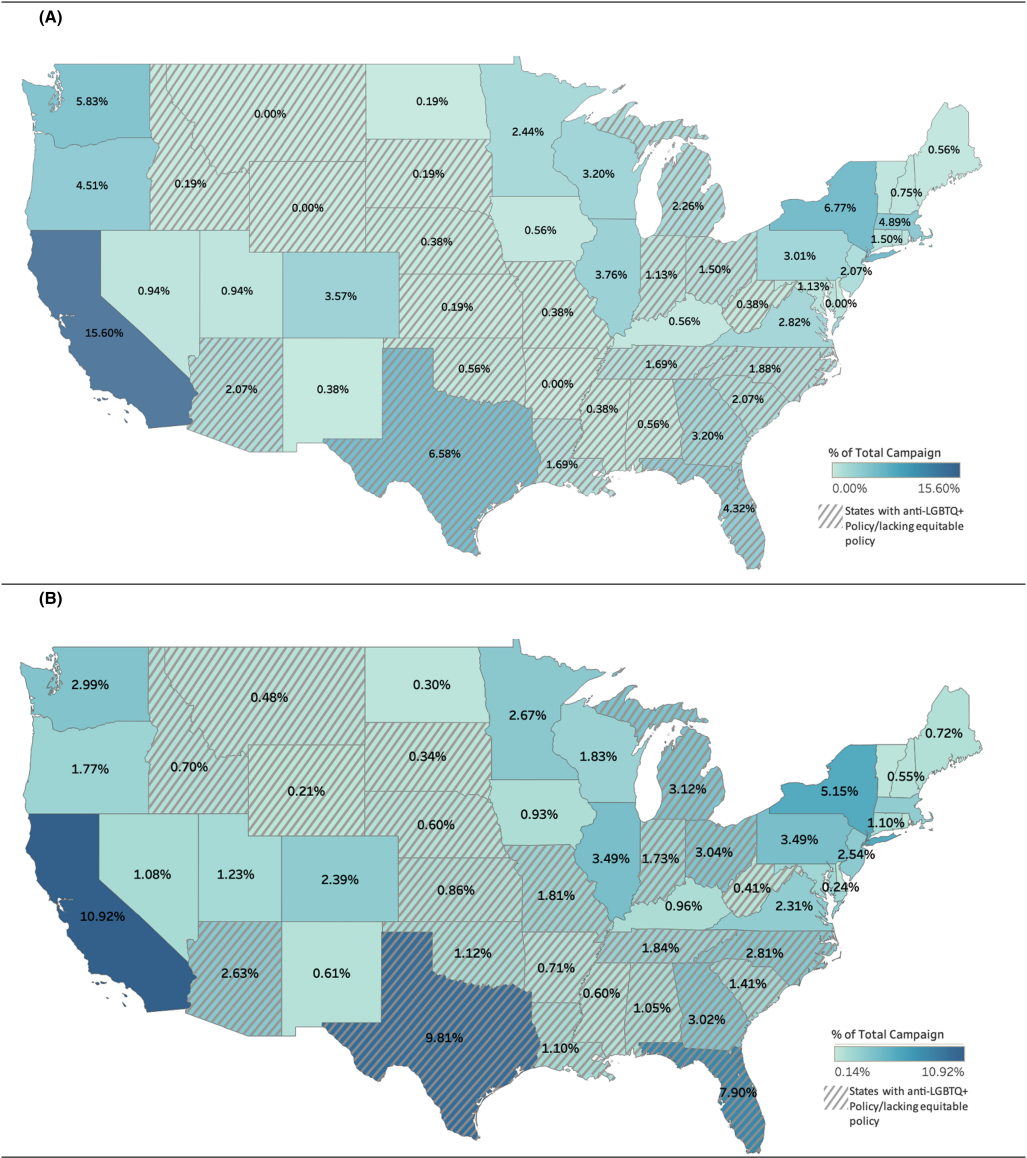
Geographical distribution of (A) LGBTQ+ and (B) non‐LGBTQ+ cancer‐related crowdfunding campaigns and LGBTQ+ inclusivity of state policy. Campaigns were started between 2014 and 2022.

In Table 1, when comparing LGBTQ+ campaigns to non‐LGBTQ+ campaigns in bivariate analyses, LGBTQ+ campaigns had a significantly higher number of donors but did not differ in goal amounts or amount raised. LGBTQ+ campaigns were more recent and had greater online reach than non‐LGBTQ+ campaigns (*p*<0.0001; Table 1). LGBTQ+ campaigns most commonly originated from the Pacific division of the United States (26.4%), while non‐LGBTQ+ campaigns most commonly originated from the South Atlantic division (20.3%). Non‐LGBTQ+ campaigns were significantly more likely to originate from states with anti‐LGBTQ+ policy/lacking equitable policy than campaigns that originated from states with some/all equitable policy (53.2% vs. 46.8%; *p*<0.0001). The geographic distribution of campaigns is visualized using two maps of the United States in Figure 1. Panel A of Figure 1 shows the percent of the total number of LGBTQ+ campaigns that originate from each state with gray stripes covering states with anti‐LGBTQ+ policy/lacking equitable policy, while Panel B of Figure 1 similarly displays the distribution of campaigns but for non‐LGBTQ+ campaigns only.

In bivariate analyses stratified by online reach and comparing campaign characteristics by LGBTQ+ status, LGBTQ+ campaigns raised significantly more money than non‐LGBTQ+ campaigns when they had minimal online reach ($2919 [IQR: 770–2392] vs. $2221 [IQR: 1170–2709], *p* = 0.0008; Table 2). However, LGBTQ+ campaigns raised significantly less money when they had low ($3134 [IQR: 2162–3725] vs. $4018 [IQR: 2555–4680], *p* < 0.0001), moderate ($6665 [IQR: 3902–8265] vs. $7364 [IQR: 4741–8799], *p* = 0.001), and high ($14,315 [IQR: 8650–18,098] vs. $16,614 [IQR: 10,375–20,296], *p* = 0.0004) online reach in comparison to non‐LGBTQ+ campaigns with low, moderate, and high online reach. There was no difference in amount raised between LGBTQ+ and non‐LGBTQ+ campaigns with viral online reach. Goal amount among campaigns with high online reach was significantly lower among LGBTQ+ compared to non‐LGBTQ+ campaigns.

**TABLE 2:** Unadjusted differences in donors, goal amount, and amount raised of cancer‐related crowdfunding campaigns by LGBTQ+ status and online reach between 2014 and 2022.

	LGBTQ+ campaigns (*N* = 535)			Non‐LGBTQ+ campaigns (*N* = 188,342)			*p*‐value
	Mean	SD	IQR	Mean	SD	IQR	
Minimal reach: 3–25 donors							
Number of donors	16	6	10–21	16	6	12–21	0.79
Goal amount ($)	15,361	20,836	5000–16,961	49,967	5,102,018	5000–16,200	0.92
Amount raised ($)	2919	12,053	770–2392	2221	1872	1170–2709	0.0008
Low reach: 26–46 donors							
Number of donors	34	5	29–38	35	6	30–40	0.20
Goal amount ($)	15,137	20,193	5400–12,194	67,430	6,859,217	5824–18,521	0.19
Amount raised ($)	3134	1887	2162–3725	4018	2606	2555–4680	<0.0001
Moderate reach: 46–87 donors							
Number of donors	64	11	54–73	63	12	53–73	0.41
Goal amount ($)	21,036	31,602	6097–24,387	80,592	7,330,673	7417–23,879	0.09
Amount raised ($)	6665	3921	3902–8265	7364	4361	4741–8799	0.001
High reach: 88–240 donors							
Number of donors	143	41	104–176	137	40	104–163	0.11
Goal amount ($)	23,361	20,950	10,800–27,000	66,861	5,834,535	11,939–34,964	0.03
Amount raised ($)	14,315	8478	8650–18,098	16,614	9577	10,375–20,296	0.0004
Viral reach: 241–15,881 donors							
Number of donors	536	364	299–626	447	491	278–465	0.22
Goal amount ($)	102,457	191,201	34,341–85,854	72,469	173,065	30,000–84,807	0.21
Amount raised ($)	50,066	32,034	31,650–60,528	49,986	44,884	28,505–58,549	0.69

*Note*: All dollar amounts were adjusted for inflation to 2022 dollars.

**FIGURE 2:**
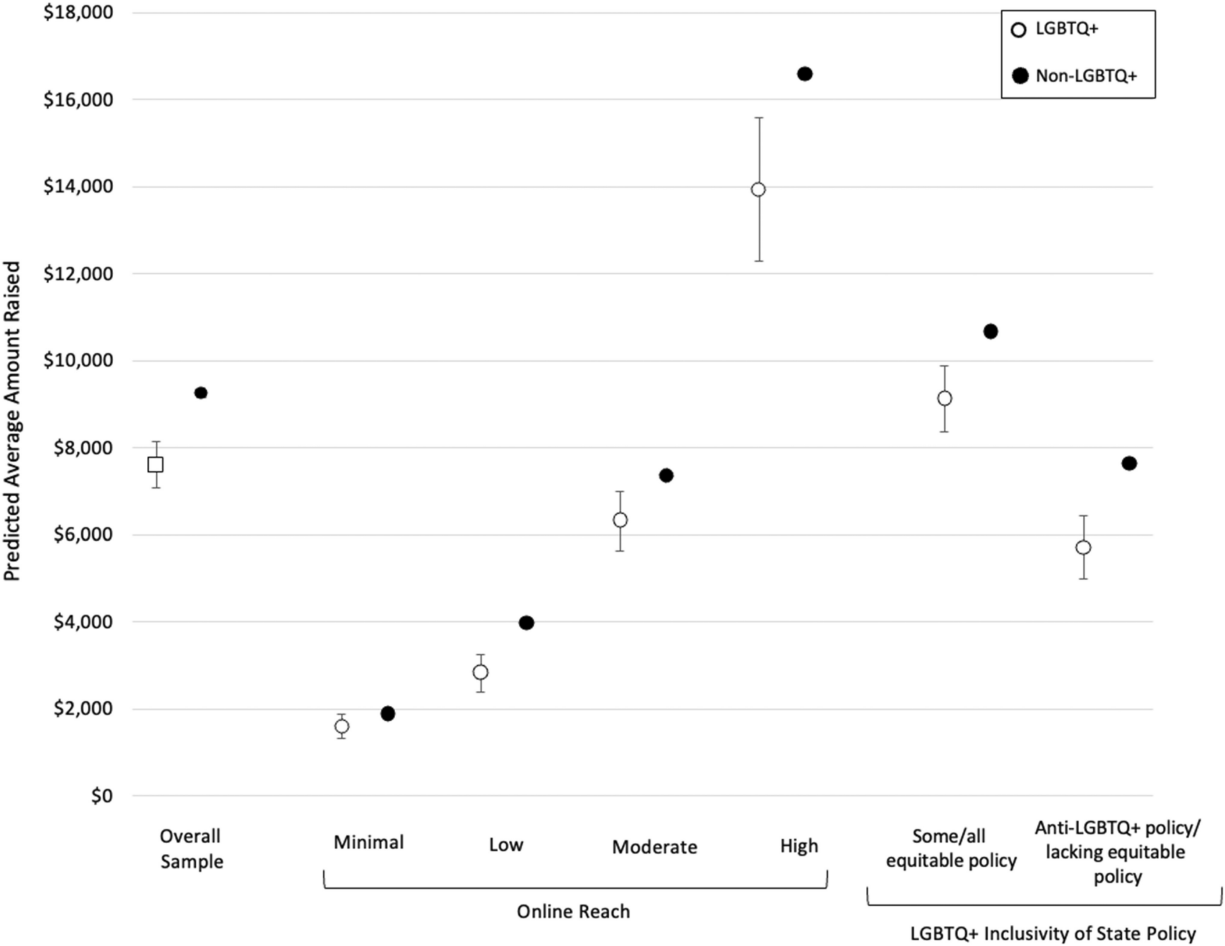
Predicted amount raised by cancer‐related crowdfunding campaigns by LGBTQ+ status: Overall and stratified by online reach and LGBTQ+ inclusivity of state policy between 2019 and 2022 HRC, Human Rights Campaign. The model controls for year campaign was initiated, geographic location, and online reach. Online reach stratified models controlled for year campaign was initiated and geographic location. Policy stratified models controlled for year campaign was initiated, geographic location, and online reach. All dollar amounts were adjusted for inflation to 2022 dollars.

In adjusted analyses among 2019–2022 campaigns, LGBTQ+ campaigns raised on average $7613 (95% CI: 7083, 8143) while non‐LGBTQ+ campaigns raised $9222 (95% CI: 9162, 9281); LGBTQ+ campaigns raised on average $1608 (95% CI: −2139, −1077) less than non‐LGBTQ+ campaigns (Table 3). When stratified by online reach, LGBTQ+ campaigns raised non‐significantly an average of $283 (95% CI: −574, 8) less than non‐LGBTQ+ campaigns at minimal online reach ($1598 [95% CI: 1307, 1888] vs. $1881 [95% CI: 1862, 1899]; Table 3). LGBTQ+ campaigns also raised significantly less money at low, moderate, and high online reach than non‐LGBTQ+ campaigns (Figure 2). LGBTQ+ campaigns with low reach raised on average $1152 (95% CI: −1589, −716) less than non‐LGBTQ+ campaigns, while LGBTQ+ campaigns at moderate and high reach raised $1050 (95% CI: −1737, −364) and $2655 (95% CI: −4312, −998) less than non‐LGBTQ+ campaigns respectively. When stratified by LGBTQ+ inclusivity of state level policy, LGBTQ+ campaigns from states with anti‐LGBTQ+ policy/lacking equitable policy raised on average $1910 (95% CI: −2640, −1182) less than non‐LGBTQ+ campaigns from the same states while LGBTQ+ campaigns in states with some or all equitable policy raised $1553 (95% CI: −2312, −793) less than non‐LGBTQ+ campaigns from the same states. No difference was observed in amount raised for campaigns with viral online reach (Table 3).

**TABLE 3:** Multivariable generalized linear models (GLM) of amount raised overall, by online reach, and by LGBTQ+ inclusivity of state policy between 2019 and 2022.

	LGBTQ+ campaigns	Non‐LGBTQ+ campaigns	Differential effect ($) [95% CI]
	Predicted amount ($) [95% CI]		
Overall sample	7613 [7083, 8143]	9222 [9162, 9281]	−1608*** [−2139, −1077]
Online reach			
Minimal (3–25 donors)	1598 [1307, 1888]	1881 [1862, 1899]	−283 [−574, 8]
Low (26–45 donors)	2822 [2386, 3257]	3974 [3942, 4006]	−1152*** [−1589, −716]
Moderate (46–87 donors)	6311 [5626, 6995]	7361 [7309, 7413]	−1050** [−1737, −364]
High (88–240 donors)	13,921 [12,269, 15,574]	16,577 [16,452, 16,702]	−2655** [−4312, −998]
Viral (241–15,881 donors)	51,620 [37,122, 66,119]	49,943 [48,822, 51,064]	1677 [−12,864, 16,218]
LGBTQ+ inclusivity of state policy			
Some/all equitable policy	9111 [8353, 9869]	10,664 [10,569, 10,758]	−1553*** [−2312, −793]
Anti‐LGBTQ+ policy/lacking equitable policy	5716 [4988, 6444]	7627 [7556, 7697]	−1910*** [−2640, −1182]

*Note*: The model controls for year campaign was initiated, geographic location, and online reach. Online reach stratified models controlled for year campaign was initiated and geographic location. Policy stratified models controlled for year campaign was initiated, geographic location, and online reach. All dollar amounts were adjusted for inflation to 2022 dollars.

* <0.05;

** <0.01;

*** <0.001.

The sensitivity analysis, which included campaigns from 2014 to 2022, revealed smaller disparities between LGBTQ+ and non‐LGBTQ+ campaigns than the main models. Specifically, the adjusted overall estimates changed substantially with LGBTQ+ campaigns raising non‐significantly less, $443 (95% CI: −958, 72) than non‐LGBTQ+ campaigns (Table 4). LGBTQ+ campaigns raised less when their online reach was low, moderate, and high, more when their online reach was minimal, and no difference at the viral reach level (Table 4). Notably, the magnitude of estimates decreased substantially when 2014–2022 data were used including LGBTQ+ campaigns. Lastly, when stratified by LGBTQ+ inclusivity of state policy, LGBTQ+ campaigns raised significantly less than non‐LGBTQ+ campaigns in states with anti‐LGBTQ+ policy or those that lacked equitable policy, but no difference was observed between LGBTQ+ and non‐LGBTQ+ campaigns in states with some or all equitable policy (Table 4).

**TABLE 4:** Sensitivity analysis: Multivariable generalized linear models (GLM) of amount raised opverall, by online reach, and by LGBTQ+ inclusivity of state policy between 2014 and 2022.

	LGBTQ+ campaigns	Non‐LGBTQ+ campaigns	Differential effect ($) [95% CI]
	Predicted amount ($) [95% CI]		
Overall sample	8817 [8302, 9332]	9259 [9215, 9304]	−443 [−958, 72]
Online reach			
Minimal (3–25 donors)	2858 [2406, 3309]	2222 [2204, 2239]	636** [184, 1087]
Low (26–45 donors)	3094 [2700, 3488]	4018 [3995, 4041]	−924*** [−1319, −529]
Moderate (46–87 donors)	6567 [5972, 7163]	7364 [7326, 7403]	−797** [−1394, −201]
High (88–240 donors)	14,144 [12,768, 15,521]	16,615 [16,519, 16,711]	−2471*** [−3851, −1091]
Viral (241–15,881 donors)	49,589 [37,191, 61,986]	49,987 [49,102, 50,872]	−399 [−12,828, 12,031]
LGBTQ+ inclusivity of state policy			
Some/all equitable policy	10,627 [9860, 11,394]	10,511 [10,441, 10,571]	115 [−652, 882]
Anti‐LGBTQ+ policy/lacking equitable policy	6376 [5732, 7019]	7834 [7782, 7886]	−1458*** [−2101, −815]

*Note*: The overall sample model controls for year campaign was initiated, geographic location, and online reach. Online reach stratified models controlled for year campaign was initiated and geographic location. Policy stratified models controlled for year campaign was initiated, geographic location, and online reach. All dollar amounts were adjusted for inflation to 2022 dollars.

* <0.05;

** <0.01;

*** <0.001.

## 4 DISCUSSION

In this national study, we identified inequity in crowdfunding among LGBTQ+ cancer campaigns compared to non‐LGBTQ+ cancer campaigns. Recent LGBTQ+ cancer campaigns on average raised $1608 less than non‐LGBTQ+ campaigns. LGBTQ+ cancer campaigns raised significantly less money than non‐LGBTQ+ campaigns in states with anti‐LGBTQ+ policy/lacking equitable LGBTQ+ policies and when campaigns had online reach that was not minimal nor viral. While crowdfunding has been a longstanding financial coping mechanism for many cancer survivors, our findings highlight LGBTQ+ inequity in crowdfunding—supporting the perspective that such a mechanism, while useful in minimizing immediate financial burden for some, largely perpetuates economic inequity among already vulnerable cancer populations.^25^

LGBTQ+ campaigns raised substantially less money than non‐LGBTQ+ campaigns overall, across most online reach levels, and by LGBTQ+ inclusivity of state policy—suggesting that online crowdfunding may perpetuate existing LGBTQ+ inequities. These findings may also be driven by existing LGBTQ+ economic inequities in social network wealth as the LGBTQ+ populations are demographically younger, more racially diverse, and more likely to live in poverty than non‐LGBTQ+ populations.^13^, ^26^ However, emerging literature suggests that LGBTQ+ cancer survivors, even when demographic factors such as race and age are considered, are more likely to experience financial burden than their non‐LGBTQ+ counterparts.^16^, ^17^ Future inquiry would benefit from using intersectional research approaches^27^ when investigating LGBTQ+ inequity in cancer‐related financial burden and the role of social network wealth.

Further, our sensitivity analysis findings indicate that LGBTQ+ campaigns raise more money than non‐LGBTQ+ campaigns at a minimal number of donors. This finding suggests that the immediate social networks of the LGBTQ+ cancer survivors whom the LGBTQ+ campaigns are raising money for may be more willing to donate higher amounts of money per person than non‐LGBTQ+ social networks. Recent social support literature suggests that LGBTQ+ communities received and valued social support from chosen family more than blood family.^28^ At the same time, LGBTQ+ campaigns ranging from low‐high online reach all raised significantly lower amounts than non‐LGBTQ+ campaigns. This finding suggests that even at low online reach, crowdfunding campaigns may have already exited the immediate social network of the fundraiser.

LGBTQ+ campaigns in states that lacked equitable LGBTQ+ policy raised significantly less than non‐LGBTQ+ campaigns at a higher magnitude than the difference between LGBTQ+ and non‐LGBTQ+ campaigns in states with some or all equitable policy. While these differences could arise due to variations in average incomes or demographics across states, anti‐LGBTQ+ attitudes and beliefs in states with anti‐LGBTQ+ policy or that lack LGBTQ+ inclusive policy likely play a large factor. Legal determinants of health influence social determinants that are dictated by laws while anti‐LGBTQ+ stigma affects health through the depletion of resources such as money, power, and prestige.^29^, ^30^ As the United States has experienced an unprecedented amount of anti‐LGBTQ+ legislation in recent years,^31^ these findings highlight the vital role that anti‐LGBTQ+ attitudes and resulting laws may play in financial burden experienced by LGBTQ+ cancer survivors, and potentially LGBTQ+ individuals who experience other diseases. Future studies should assess the causality of how policy changes impact the financial well‐being of LGBTQ+ cancer survivors and how health systems can better center LGBTQ+ populations to minimize the negative impacts of anti‐LGBTQ+ policy in their states.

Our findings are consistent with earlier research in medical crowdfunding that suggests campaigns raising money for racial minorities and women experience systematic inequities in online crowdfunding success.^32^, ^33^ LGBTQ+ crowdfunding studies outside of a cancer context have primarily focused on access to high‐cost gender‐affirming care that is often not covered by health insurance.^34^ Such studies have revealed that while crowdfunding may provide financial access to needed care for some transgender individuals it may also perpetuate existing inequities.^34^ Furthermore, literature on cancer crowdfunding inequities suggests that crowdfunding can be beneficial in reducing costs for the wealthiest patients and those with access to networks of wealth while leaving those from the most vulnerable socioeconomic status behind.^9^, ^25^

While we know of no current policy nor procedural steps to ameliorate this inherent crowdfunding inequity, what is certain is that such inequity is not due to the individual user's fault, but rather the systemic wealth inequalities across social networks, limiting the crowdfunding potential for many.^25^ The findings of this study are consistent with the existing literature, suggesting that when cancer patients are forced to rely on crowdfunding to afford their medical and non‐medical costs, such mechanisms mirror systems of oppression that drive economic inequity in the population.^9^, ^25^, ^35^, ^36^ Crowdfunding is not a solution to cancer‐related financial burden but rather one source of financial support used by patients in an often unaffordable healthcare system.

## 5 LIMITATIONS

Our study has limitations. First, access to demographic and clinical factors known to be associated with financial burden were unavailable. Although we included all active campaigns dating back to 2014, this analysis is limited to currently active campaigns. We were unable to control for cost of living by state, however, LGBTQ+ campaigns were more likely to originate from coastal states with higher cost of living—likely down biasing the magnitude of our estimates. While we limited our sample to the US based campaigns, it is possible that recipients of the campaign live in other countries. Further, LGBTQ+ campaigns from states with anti‐LGBTQ+ policy or lacking equitable policy may have been deleted at higher rates due to inhospitable cultural climate, which may have provided a shorter timeline to raise needed funds. This unobserved influence of deletions may have biased estimates, but directionality cannot be ascertained without additional research as LGBTQ+ campaigns that raised a high amount may have been quickly deleted just as often as LGBTQ+ campaigns that raised a lower amount. Due to methodological and dataset limitations, we were unable to disaggregate LGBTQ+ identities to explore differences by campaigns raising funds for sexual versus gender minority LGBTQ+ cancer survivors. Lastly, identification of LGBTQ+ status in this study relied on disclosure of sexual orientation or gender identity through the campaign description. However, anti‐LGBTQ+ attitudes and stigma may have made LGBTQ+ persons reticent to disclose their identity online, particularly if they felt doing so would threaten the success of their crowdfunding. Thus, we may have underestimated the impact of LGBTQ+ status on crowdfunding outcomes. Overall, the innovative methodology and results of this study contribute novel findings to the LGBTQ+ cancer financial burden literature.

## 6 CONCLUSION

We found that LGBTQ+ cancer crowdfunding campaigns raised significantly less money overall, at most levels of online reach, and in states with anti‐LGBTQ+ policy/lacking equitable policy for LGBTQ+ populations. These novel and important findings add to the growing LGBTQ+ cancer‐related financial burden literature. Our results highlight the substantial impact that anti‐LGBTQ+ stigma, attitudes, and policy may have on the economic well‐being of LGBTQ+ populations. More research is needed to fully understand the financial burden and cost‐coping behaviors, such as crowdfunding, among economically unstable populations such as LGBTQ+ cancer survivors.

## AUTHOR CONTRIBUTIONS

**Austin R. Waters:** Conceptualization (equal); data curation (equal); formal analysis (lead); funding acquisition (supporting); investigation (equal); methodology (lead); project administration (equal); validation (equal); visualization (equal); writing – original draft (lead); writing – review and editing (equal). **Caleb W. Easterly:** Data curation (equal); formal analysis (equal); investigation (equal); methodology (equal); validation (equal); writing – review and editing (equal). **Cindy Turner:** Data curation (equal); investigation (equal); methodology (equal); project administration (lead); validation (equal); writing – review and editing (equal). **Lauren Ghazal:** Data curation (equal); formal analysis (equal); methodology (equal); validation (equal); visualization (equal); writing – review and editing (equal). **Ida Tovar:** Data curation (equal); investigation (equal); methodology (equal); validation (equal); writing – review and editing (equal). **Megan Mulvaney:** Data curation (equal); investigation (equal); methodology (equal); validation (equal); writing – review and editing (equal). **Matt Poquadeck:** Data curation (equal); investigation (equal); methodology (equal); validation (equal); writing – review and editing (equal). **Stephen A. Rains:** Funding acquisition (equal); investigation (equal); methodology (equal); supervision (equal); writing – review and editing (equal). **Kristin G. Cloyes:** Funding acquisition (equal); investigation (equal); methodology (equal); supervision (equal); writing – review and editing (equal). **Anne C. Kirchhoff:** Funding acquisition (equal); investigation (equal); methodology (equal); supervision (equal); writing – review and editing (equal). **Erin E. Kent:** Investigation (equal); methodology (equal); supervision (equal); writing – review and editing (equal). **Echo L. Warner:** Conceptualization (equal); data curation (equal); formal analysis (equal); funding acquisition (equal); investigation (equal); methodology (equal); project administration (equal); resources (equal); supervision (equal); validation (equal); visualization (equal); writing – review and editing (equal).

## FUNDING INFORMATION

Research reported in this publication was supported by the National Cancer Institute of the National Institutes of Health under Award Number P30CA042014‐31S3. This content is solely the responsibility of the authors and does not necessarily represent the official views of the National Institutes of Health. Austin R. Waters is supported by the National Cancer Institute's National Research Service Award sponsored by the Lineberger Comprehensive Cancer Center at the University of North Carolina (T32 CA116339).

## CONFLICT OF INTEREST STATEMENT

The authors declare that there are no conflicts of interest or financial disclosures related to this research.

## Data Availability

The data used in this study are available upon reasonable request to the corresponding author, ARW.
